# Seroepidemiology of *Helicobacter pylori* Infection in Tepehuanos Aged 15 Years and Older in Durango, Mexico

**DOI:** 10.1155/2013/243246

**Published:** 2013-03-20

**Authors:** Cosme Alvarado-Esquivel

**Affiliations:** Faculty of Medicine and Nutrition, Juárez University of Durango State, Avenida Universidad S/N, 34000 Durango, DGO, Mexico

## Abstract

This study aimed to determine the seroepidemiology of *Helicobacter pylori* infection in Tepehuanos (an indigenous ethnic group living in rural Mexico). The prevalence of anti-*Helicobacter pylori* IgG antibodies was examined in 156 Tepehuanos in Durango State, Mexico, using an enzyme-linked immunoassay. In addition, sociodemographic, clinical, and behavioral characteristics of Tepehuanos associated with seropositivity were investigated. In total, 103 (66%) of the 156 participants (mean age 31.03  ±  16.71 years) had *Helicobacter pylori* IgG antibodies. Fifty-four (52.4%) of the 103 seropositive individuals had *Helicobacter pylori* IgG antibody levels higher than 100 U/mL. Males and females had comparable seroprevalence of *Helicobacter pylori* infection and *Helicobacter pylori* IgG antibody levels. The seroprevalence was significantly higher in women with pregnancies than those without this obstetric characteristic. Logistic regression showed that *Helicobacter pylori* infection was positively associated with low education (OR = 3.37; 95% CI: 1.13–10.00; *P* = 0.02) and laborer occupation (OR = 2.71; 95% CI: 1.14–6.42; *P* = 0.02). This is the first report of seroprevalence and contributing factors for *Helicobacter pylori* infection in Tepehuanos and of the association of *Helicobacter pylori* infection with laborer occupation. Results warrants further research.

## 1. Introduction

The spiral-shaped and flagellated bacillus *Helicobacter pylori* causes infections in humans worldwide [1]. Estimates indicate that *H. pylori* is currently infecting approximately one half of the world's population [1, 2]. Although most infections with *H. pylori* are asymptomatic, a severe gastric disease including chronic gastritis, peptic ulcer, gastric mucosa-associated lymphoid tissue lymphoma, and gastric cancer may occur in some individuals [1–4]. According to the current knowledge, dissemination of *H. pylori* might occur from person to person [5] and by oral-oral or oral-fecal routes [6]. Infections with *H, pylori* might also occur by drinking contaminated water [6, 7]. The seroprevalence of *H. pylori* varies substantially among countries being significantly higher in developing countries than in developed countries [8]. The prevalence of infection also varies among geographical regions and ethnic groups [6]. Very little knowledge about the epidemiology of *H. pylori *infection in Mexico exists. There is a lack of information about *H. pylori* infection in Tepehuanos (an ethnic group in northern Mexico). Tepehuanos consist of indigenous people living mostly in little remote rural communities. A cross-sectional study was performed to determine the seroprevalence of anti-*H. pylori *antibodies in Tepehuanos in Durango State, Mexico. In addition, sociodemographic, clinical, and behavioral characteristics associated with *H. pylori *seropositivity in Tepehuanos were also investigated. 

## 2. Materials and Methods

### 2.1. Study Design and Study Population

Through a cross-sectional study using serum samples from a recent *Toxoplasma gondii* serosurvey [9], 156 Tepehuanos living in Durango State, Mexico, were studied. In the previous study, the purpose of sera collection was to determine the seroepidemiology of *Toxoplasma gondii* in Tepehuanos, and sera were collected from January 2010 to March 2011. Inclusion criteria for voluntary participation of the subjects were (1) subjects of Tepehuano ethnicity, (2) 15 years and older, (3) any gender, (4) any occupation, and (5) any socioeconomic level. 

### 2.2. Sociodemographic, Clinical, and Behavioral Data

Characteristics of the participants were obtained with the aid of a standardized questionnaire. Sociodemographic data including age, gender, birth place, residence, educational level, occupation, and socioeconomic status were obtained from all participants. Socioeconomic status in Tepehuanos was ranked by themselves according to their own perception. Clinical data explored in Tepehuanos included the presence of any disease, presence or history of gastritis, peptic ulcer, gastric cancer, and lymphadenopathy. Clinical data was obtained from the participants, and a diagnosis of diseases was based on previous medical consultations. Confirmation of clinical data by means of further diagnostic procedures was beyond the scope of the present survey. For women, obstetric data was also recorded. Behavioral data included animal contacts, foreign travel, consumption of meat, unpasteurized milk, unwashed raw vegetables or fruits, or untreated water, frequency of eating away from home (in restaurants or fast food outlets), contact with soil (gardening or agriculture), and type of floorings at home. 

### 2.3. Serological Examination for *H. pylori* Antibodies

Serum samples were obtained from all participants and kept frozen at –20°C until examined. Serum samples were analyzed through qualitative and quantitative methods for anti-*H. pylori* IgG antibodies with a commercially available enzyme immunoassay kit, that is, “Anti-*H. pylori* IgG AccuBind ELISA” (Monobind Inc., Lake Forest, CA). Anti-*H. pylori* IgG antibody levels were expressed as units (U)/mL, and a result greater than 20 U/mL was considered positive. Test was performed following the instructions of the manufacturer.

### 2.4. Statistical Analysis

 The statistical analysis was performed with the aid of the software Epi Info version 3.5.4 and SPSS version 15.0. For calculation of the sample size the following values were used: a reference seroprevalence of 66% [10] as expected frequency of the factor under study, 7000 as the size of population from which the sample was selected, a worst acceptable result of 58.5%, and a confidence level of 95%. The result of the calculation was 150 subjects. The Pearson chi-square test and the Fisher exact test (when values were less than 5) were used for comparison of the frequencies among groups. Age among groups was compared by the Student's *t*-test. Bivariate and multivariate analyses were used to assess the association between the characteristics of the subjects and *H. pylori* seropositivity. As a criterion for inclusion of variables in the multivariate analysis, variables with *P* < 0.20 obtained in the bivariate analysis were considered. Odds ratio (OR) and 95% confidence interval (CI) were calculated by multivariate analysis using logistic regression with the Enter method. A *P* value of < 0.05 was considered statistically significant.

### 2.5. Ethical Considerations

This study was approved by the Ethical Committee of the Instituto de Seguridad y Servicios Sociales de los Trabajadores del Estado in Durango City. Only archival serum samples and questionnaires from the previous survey [9] were used in the present study. However, in the previous survey, the purpose and procedures of the studies were explained to all participants, and a written informed consent was obtained from each participant. The previous survey was approved by an Institutional Ethical Committee.

## 3. Results

Of the 156 Tepehuanos studied, 103 (66.0%) were positive and 53 (34%) were negative for *H. pylori* IgG antibodies. General sociodemographic characteristics of the 156 Tepehuanos studied are shown in Table 1. Most participants were born in Durango State; their mean age was 31.03 ± 16.71 years (range 15–89 years). The seroprevalence of *H. pylori* infection was not influenced by gender, birth place, residence, or socioeconomic status. In contrast, the seroprevalence of *H. pylori* infection varied significantly with age, educational level, and occupation. Increased seroprevalence in Tepehuanos was found in the age groups of 41–50 (100%) and 61–70 (100%) years old and in those with low education (up to 6 years of education) (82.4%). In addition, laborer Tepehuanos (employees, construction workers, agriculturists, etc.) had a significantly (*P* = 0.0001) higher seroprevalence than nonlaborer Tepehuanos (housewives, students, or neither). Of the 103 *H. pylori* IgG positive participants, 54 (52.4%) had IgG levels higher than 100 U/mL, 27 (26.2%) between 51 to 100 U/mL, and 22 (21.4%) from 21 to 50 U/mL. Levels of anti-*H. pylori* IgG antibodies were similar in men and women (*P* = 0.30). 

With respect to clinical data, there were two Tepehuanos suffering from gastritis, and both were positive for *H. pylori* antibodies. One of them has had lymphadenopathy. The frequency of gastritis in *H. pylori* seropositive (2/103) and *H. pylori* seronegative (0/53) Tepehuanos was similar (*P* = 0.43). No cases of peptic ulcer or gastric cancer among Tepehuanos were found. Ill participants suffered from a number of diseases including arthritis, back pain, eye disease, epilepsy, upper respiratory tract infections, and others. Clinical data was similar among *H. pylori* positive and *H. pylori* negative individuals. In women, the seroprevalence of* H. pylori* infection was significantly (*P* = 0.02) higher in women with pregnancies (35/48: 72.9%) than those without such history (19/38: 50.0%). The seroprevalence of *H. pylori* infection was similar (*P* = 0.60) in pregnant and nonpregnant women (1/2 and 53/84, resp.). The seroprevalence was similar in women who have had cesarean sections, deliveries, and abortions than those without such obstetric characteristics. 

Regarding behavioral characteristics, three variables showed *P*  values < 0.20 by bivariate analysis: consumption of meat (*P* = 0.03), frequency of meat consumption (*P* = 0.04), and frequency of eating away from home (*P* = 0.06). Other behavioral characteristics including animal contacts, foreign travel, consumption of unpasteurized milk, unwashed raw vegetables or fruits, or untreated water, contact with soil, and type of floorings at home showed *P* values higher than 0.20 in the bivariate analysis.  Table 2 shows the results of the bivariate analysis of behavioral characteristics and *H. pylori* seroprevalence. Further analysis using logistic regression of sociodemographic and behavioral characteristics of Tepehuanos showed that *H. pylori* infection was positively associated with low education (OR = 3.37; 95% CI: 1.13–10.00; *P* = 0.02) and laborer occupation (OR = 2.71; 95% CI: 1.14–6.42; *P* = 0.02) (Table 3). 

## 4. Discussion

 The 66% seroprevalence of *H. pylori* infection found in Tepehuanos in Durango, Mexico, is similar to the mean national seroprevalence (66%) reported in Mexico [10]. However, this comparison should be interpreted with care, since different test methods in the studies were used. A commercial ELISA was used in the present study, while a homemade ELISA was used in the national survey. The national survey in Mexico [10] did not provide specific seroprevalence in Durango. However, the seroprevalence found in Tepehuanos is higher than a 50.7% seroprevalence found in Mennonites (an ethnic group of German descent living in rural communities) in Durango [11]. In both studies in ethnic groups in Durango the same test (commercial ELISA) was used. In an international context, the seroprevalence of *H. pylori* infection in Tepehuanos in Durango is lower than the estimated 80%–90% seroprevalence of *H. pylori* infection in developing countries [8]. 

With respect to the sociodemographic characteristics of Tepehuanos, seropositivity to *H. pylori* was found even in the youngest participants (55.1% in ages of 15–30 years old), and some older groups (41–50 and 61–70 years old) showed a 100% seroprevalence of *H. pylori* infection. The former suggests an early exposure to *H. pylori* in the studied population, and the latter follows a typical increase in the frequency of *H. pylori* infection in humans as reported elsewhere [5, 10, 12]. In the present study, multivariate analysis of sociodemographic and behavioral characteristics of Tepehuanos showed that seropositivity to *H. pylori* was associated with low education (up to 6 years of education) (OR = 3.37; 95% CI: 1.13–10.00; *P* = 0.02) and laborer occupation (OR = 2.71; 95% CI: 1.14–6.42; *P* = 0.02). The finding of an association between *H. pylori* infection and low educational level in Tepehuanos agrees with the data found in the national survey in Mexico [10]. On the other hand, the finding that laborer Tepehuanos showed a significantly higher seroprevalence of *H. pylori* than nonlaborers is intriguing. The variable age was not responsible for the difference in the seroprevalence among laborer and nonlaborer Tepehuanos, since age adjustment was included in the multivariate analysis. To my knowledge, there are not any previous reports of an association of laborer occupation with *H. pylori* seropositivity. It is not clear why laborer Tepehuanos had a higher *H. pylori* seroprevalence than nonlaborers. It is possible that laborer Tepehuanos are in closer contact with the *H. pylori* source of infection than nonlaborer Tepehuanos. Other sociodemographic and behavioral characteristics explored in Tepehuanos were not associated with *H. pylori* seropositivity. The socioeconomic status did not influence the seroprevalence of *H. pylori* infection in Tepehuanos. This finding conflicts with those found in other studies [5, 10] where *H. pylori* infection was associated with a low socioeconomic status. Consumption of meat has been associated with a significant increase of anti-*H. pylori* IgM antibodies in Kenyan children [13]. In addition, *H. pylori* infection has been related with meat consumption in children in Mexico [14]. However, the associations of *H. pylori* seropositivity with meat consumption and frequency of meat consumption obtained in the bivariate analysis in the present study did not resist the multivariate analysis. Further research about the association of *H. pylori* infection with meat consumption using a larger sample size population is needed. 

Of the clinical characteristics explored, there was no difference in the frequency of gastritis or other clinical data among *H. pylori* positive and *H. pylori* negative Tepehuanos. There were only 2 gastritis cases, and both were positive for *H. pylori*. Therefore, *H. pylori*-associated gastritis exists among Tepehuanos, and further research to evaluate the impact of *H. pylori* on the health of Tepehuanos is needed. In the present study, the seroprevalence of *H. pylori* infection was significantly higher in women who have had pregnancies than those without this obstetric history. This finding agrees with that found in a study of pregnant women in Israel [15] where researchers found that women positive for *H. pylori* had more prior pregnancies than *H. pylori* negative women. The higher seroprevalence of *H. pylori* infection in women with pregnancies than those without pregnancies found in the present study was likely due to difference in age among the groups. The mean age in women with pregnancies (36.3 ± 14.8 years old) was significantly higher than that (18.6 ± 5.7 years old) in women without pregnancies (*P* < 0.000001).

This is the first report of seroprevalence and contributing factors for *Helicobacter pylori *infection in Tepehuanos and of the association of *Helicobacter pylori* infection with laborer occupation. Results warrants further research.

## Figures and Tables

**Table 1:** Sociodemographic characteristics of Tepehuanos and seroprevalence of *H. pylori* infection.

Characteristic	No. of subjects tested^a^	Prevalence of *H. pylori* infection		*P* value
		No.	%	
Gender				
Male	68	48	70.6	0.29
Female	88	55	62.5	
Age groups (years)				
15–30	98	54	55.1	0.002
31–40	19	16	84.2	
41–50	13	13	100.0	
51–60	15	11	73.3	
61–70	6	6	100.0	
71–89	4	2	50.0	
Birth place				
Durango State	153	102	66.7	0.62
Other Mexican state	2	1	50.0	
Residence area				
Urban	13	8	61.5	0.74
Rural	141	93	66.0	
Educational level				
Up to 6 years of education	74	61	82.4	0.00001
7 or more years of education	81	41	50.6	
Occupation				
Laborer^b^	68	56	82.4	0.0001
Nonlaborer^c^	88	47	53.4	
Socioeconomic level				
Low	147	95	64.6	0.26
Medium	8	7	87.5	

^a^Subjects with available data.

^b^Employee, construction worker, business, factory worker, others.

^c^Housewife, student, or neither.

**Table 2:** Bivariate analysis of selected behavioral characteristics of Tepehuanos and seroprevalence of *H. pylori* infection.

Characteristic	No. of subjects tested^a^	Prevalence of *H. pylori* infection		*P* value
		No.	%	
Cats at home				
Yes	80	52	65	0.78
No	76	51	67.1	
Raising animals^b^				
Yes	130	86	66.2	0.94
No	26	17	65.4	
Eating outside home				
Never	33	26	78.8	0.06
From 1 to 10 times a year	99	59	59.6	
More than 10 times a year	22	17	77.3	
Meat consumption				
Yes	153	103	67.3	0.03
No	3	0	0	
Frequency of meat consumption				
Never	3	0	0	0.04
Up to 3 times a week	148	100	67.6	
4–7 times a week	4	3	75	
Raw cow milk consumption				
Yes	106	69	65.1	0.59
No	49	34	69.4	
Unwashed raw vegetables				
Yes	62	40	64.5	0.67
No	93	63	67.7	
Unwashed raw fruits				
Yes	85	56	65.9	0.86
No	70	47	67.1	
Untreated water				
Yes	128	84	65.6	0.63
No	27	19	70.3	
Traveled abroad				
Yes	7	5	71.4	1
No	149	98	65.8	
National trips				
Yes	60	42	70	0.38
No	95	60	63.2	
Soil contact				
Yes	142	95	66.9	0.76
No	13	8	61.5	
Floor at home				
Ceramic	21	14	66.7	0.73
Concrete	57	40	70.2	
Soil	77	49	63.6	

^a^Sums may not add up to 156 because of missing values.

^b^Raising of any kind of animals.

**Table 3:** Multivariate analysis of selected characteristics of Tepehuanos and their association with *H. pylori* infection.

Characteristic	Odds ratio	95% confidence interval	*P* value
Age	1	0.96–1.03	0.98
Low education	3.37	1.13–10.00	0.02
Laborer	2.71	1.14–6.42	0.02
Eating outside home	0.94	0.46–1.93	0.88
Meat consumption	—	—	0.99
Frequency of meat consumption	1.06	0.08–13.15	0.95
